# Brønsted acid-promoted azide–olefin [3 + 2] cycloadditions for the preparation of contiguous aminopolyols: The importance of disiloxane ring size to a diastereoselective, bidirectional approach to zwittermicin A

**DOI:** 10.3762/bjoc.6.138

**Published:** 2010-12-20

**Authors:** Hubert Muchalski, Ki Bum Hong, Jeffrey N Johnston

**Affiliations:** 1 Vanderbilt University and Vanderbilt Institute of Chemical Biology, Nashville, TN 37235, United States

**Keywords:** azide, bidirectional synthesis, cycloaddition, dialkoxydisiloxane, TIPDS, triazoline, triflic acid, zwittermicin A

## Abstract

We report the first study of substrate-controlled diastereoselection in a double [3 + 2] dipolar cycloaddition of benzyl azide with α,β-unsaturated imides. Using a strong Brønsted acid (triflic acid) to activate the electron deficient imide π-bond, high diastereoselection was observed provided that a 1,1,3,3-tetraisopropoxydisiloxanylidene group (TIPDS) is used to restrict the conformation of the central 1,3-*anti* diol. This development provides a basis for a stereocontrolled approach to the aminopolyol core of (−)-zwittermicin A using a bidirectional synthesis strategy.

## Introduction

**Scheme 1:**
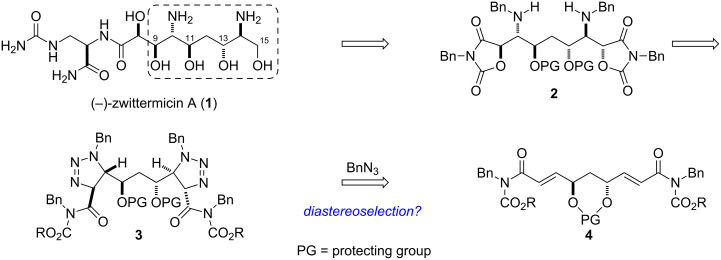
Retrosynthetic analysis outlining the stereocontrolled construction of the aminopolyol core of (−)-zwittermicin A using an azide–olefin double cycloaddition.

Structural motifs such as 1,2-aminoalcohol, 1,2- and 1,3-diol are very prevalent features in natural products, especially polyketides. The structures of some of these, such as sorbistin A1 [1] or zwittermicin A [2], contain mostly aminopolyol moieties. Aminoalcohol and diol motifs are often constructed via alkene functionalization such as aminohydroxylation [3] and dihydroxylation [4] reactions, or by methods that forge the carbon–carbon bond such as the glycolate Mannich reaction [5]. Recently, we developed a Brønsted acid-promoted azide–olefin reaction as an alternative to metal catalyzed aminohydroxylations [6–8]. Triflic acid-promoted reaction of an alkyl azide with an α,β-unsaturated imide delivers a formal *anti*-aminohydroxylation product. We wondered whether azide–olefin functionalization could be used to prepare the complex aminopolyol core of zwittermicin A [9–12]. We were particularly intrigued by the possibility of a substrate controlled *anti*-diastereoselective azide–olefin reaction performed in a bidirectional fashion [13–14] to establish the requisite stereocenters of the C9–C15 *C*_2_ symmetric core of the natural product [11] as outlined in Scheme 1.

Diastereoselective functionalization of the alkene of chiral allylic alcohols and ethers can be highly efficient, and the substrates are often easily accessible. The use of steric effects to achieve facial discrimination can be achieved by the introduction of a silyl group. Although several reports of diastereoselective azide–olefin cycloaddition reactions exist, they rely on intramolecular azide delivery under thermal conditions [15–21]. Examples of substrate control in an acid-catalyzed intermolecular reaction of azides with alkenes are limited [22]. We report our initial study of the intermolecular, diastereoselective sequence of two [3 + 2] cycloaddition reactions promoted by triflic acid where the large 1,3-diol protecting group – the 1,1,3,3-tetraisopropoxydisiloxanylidene group (TIPDS) – plays a crucial role in facial discrimination.

## Results and Discussion

Our approach to the preparation of enantiomerically pure (−)-zwittermicin A (**1**) is based on a short synthesis of the C9–C15 aminopolyol core that takes advantage of its underlying *C*_2_ symmetry, as outlined in Scheme 1. Desymmetrization and functionalization of the bis(oxazolidine dione) **2** provides us with a foundation for the synthesis of **1** and would arise from the acid-promoted fragmentation of 2,3-*anti* / 2’,3’-*anti* bis(triazoline) **3** (Scheme 1). Compound **3** would be assembled in a sequential substrate-controlled intermolecular cycloaddition between imide **4** and benzyl azide.

We envisioned that the facial discrimination could be provided by a large alcohol protecting group at the central *anti*-1,3-diol (C11 and C13 in zwittermicin A). Our study of the diastereoselective reaction of benzyl azide with the bis(imide) is presented in Table 1. We began with a thermal reaction of the bis(imide) **5** that used the common di(*tert*-butylsilyl) functionality as a directing group [23–25]. After 45 min at 100 °C under microwave irradiation in neat benzyl azide [26], all three possible stereoisomers formed non-selectively (1:2:1 ratio). The desired 2,3-*anti* diastereomer **10a** was separated and the relative stereochemistry was assigned through a spectroscopic study (NOESY, see Supporting Information File 1). Although the overall yield was satisfactory, purification of the desired 2,3-*anti /* 2’,3’-*anti* diastereomer **10a** was tedious.

**Table 1:** Substrate-controlled double [3 + 2] cycloaddition.

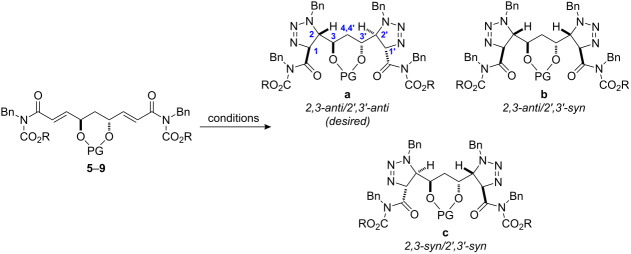							

Entry	Alkene	PG	R	Conditions^a^	Product	**a**:**b**:**c**^b^	Yield (%)^c^

1	**5**	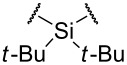	Me	A	**10**	1:2:1	72
2	**5**	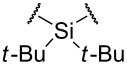	Me	B	**10**	1:2.5:5.4	67
3	**6**	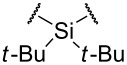	*i-*Pr	B	**11**	1:9:9	54
4	**7**	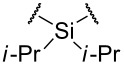	*i-*Pr	B	**12**	ND^d^	7
5	**8**	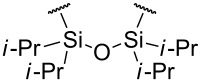	Me	B	**13**	18:1:1	27
6	**9**	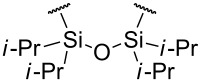	*i-*Pr	B	**14**	18:1:1	79

^a^Conditions A: BnN_3_ (excess), microwave 100 °C, 1 h; Conditions B: TfOH (5 equiv), BnN_3_ (10 equiv) MeCN [0.2 M], −20 °C, 18 h. ^b^Ratio of products was measured using the ^1^H NMR of the crude reaction mixture. ^c^Combined isolated yield. ^d^ND = not determined due to signal overlap in ^1^H NMR.

We then turned our attention to triflic acid-promoted triazoline formation. When imide **5** was reacted with BnN_3_ in the presence of triflic acid at −20 °C in acetonitrile, again, all three bis(triazolines) were isolated in 67% yield (Table 1, entry 2). In this experiment, however, the 2,3-*syn* diastereomer **10b** was slightly favored and the desired **10a** formed as a minor product. We found that a change of the ester group of the carbamate functionality from Me to *i*-Pr slightly improves selectivity. However, compound **6** led to mostly the undesired bis(triazolines) **11b** and **11c** (entry 3) in 54% combined yield whilst the desired product **11a** was present in only trace amounts. Bis(imide) **7** with a smaller diisopropyl silyl ring decomposed under the reaction conditions and gave the mixture of triazolines in only 7% yield, the ratio of which could not be determined from the ^1^H NMR spectrum (Table 1, entry 4).

**Scheme 2:**
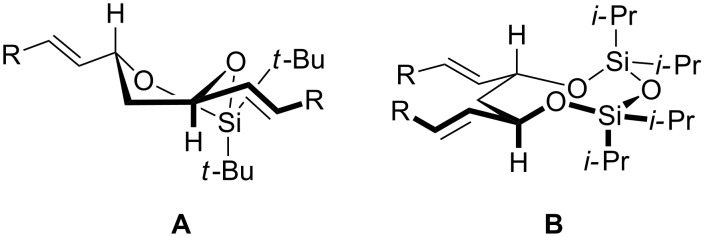
Depictions of the likely major conformations of the siloxane (A) and disiloxane (B) rings.

Our original expectation was that the siloxane protected 1,3-*anti*-diol would assume a twist-boat conformation in order to maintain its two alkene substituents in a *pseudo*-equatorial arrangement. We reasoned that expansion of the ring from six to eight members through the formation of a disiloxanylidene derivative might better achieve this goal by providing greater flexibility around the oxygen-substituted edge (Scheme 2).

The 8-membered ring methyl carbamate **8** incorporating a tetraisopropoxydisiloxanylidene group [24–25] (TIPDS) was prepared. Not only did bis(imide) **8** provide the bis(triazoline) with high diastereoselection (Table 1, entry 5), it favored the desired *anti*,*anti* **13a** (30% yield). Introduction of the isopropyl carbamate in bis(imide) **9** led to a significant increase in the yield of the 2,3-*anti-*bis(triazoline) **14** (79%) without loss of diastereoselection (Table 1, entry 6).

**Scheme 3:**
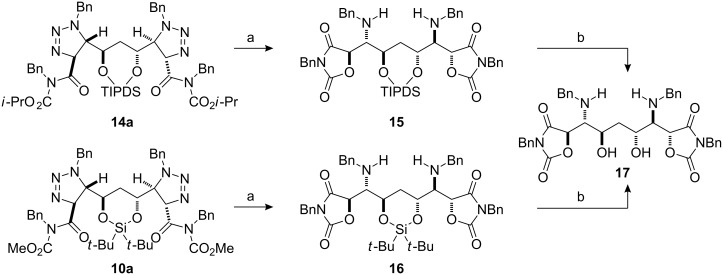
Confirmation of the relative stereochemistry of bis(triazoline) **14a**. (a) TfOH, CH_3_CN, rt, 18 h; (b) HF·pyridine, THF, 0 °C to rt, 1 h.

Due to the flexibility of the disiloxane ring we were unable to determine reliably the relative stereochemistry of **13** or **14** by NOE. However, bis(triazolines) **10a** and **14a** could be converted to the corresponding bis(oxazolidine diones) by treatment with triflic acid at room temperature (Scheme 3). The silyl protecting groups were removed with HF·pyridine in THF, and **15** and **16** converted to the same 1,3-diol **17** (Scheme 3).

## Conclusion

In summary, this first study of the substrate-controlled diastereoselective addition of benzyl azide to an unsaturated bis(imide) has demonstrated that high diastereoselection is possible using an *anti*-1,3-diol scaffold. However, it is important to protect this diol as an 8-membered dialkoxydisiloxane instead of a more traditional 6-membered dialkoxysilane. The *anti*,*anti*-selectivity observed in this transformation provides a foundation for the straightforward preparation of the aminopolyol backbone of (−)-zwittermicin A using a bidirectional chain functionalization strategy.

## Supporting Information

File 1 Experimental procedures, ^1^H and ^13^C NMR spectra.
