# Supplementary material for: Brønsted acid-promoted azide–olefin [3 + 2] cycloadditions for the preparation of contiguous aminopolyols: The importance of disiloxane ring size to a diastereoselective, bidirectional approach to zwittermicin A
Source: Beilstein J Org Chem. 2010 Dec 20;6:1206–10. doi: 10.3762/bjoc.6.138 (PMC3028999; doi:10.3762/bjoc.6.138)
Supplement: File 1 — Experimental procedures, 1H and 13C NMR spectra. [file Beilstein_J_Org_Chem-06-1206-s001.pdf]

Supporting Information
for
Brønsted acid-promoted azide–olefin [3 + 2] cycloadditions for the preparation of contiguous aminopolyols: The importance of disiloxane ring size to a diastereoselective, bidirectional approach to zwittermicin A.

Hubert Muchalski, Ki Bum Hong and Jeffrey N. Johnston*[1]

Address: [1]Vanderbilt University and Vanderbilt Institute of Chemical Biology, Nashville, TN 37235, United States

Email: Jeffrey N. Johnston - jeffrey.n.johnston@vanderbilt.edu

*Corresponding Author

Experimental procedures, ^{1}H and ^{13}C NMR spectra

Contents

General Experimental Details **2**
Triazoline **10** 2
General Procedure for Triflic Acid-Promoted Bis(triazoline) Formation 2
Triazoline **11** 3
2,3-*anti*/2′,3′-*anti* Bis(triazoline) **13a** 3
2,3-*anti*/2′,3′-*anti* Bis(triazoline) **14a** 3
Bis(oxazolidine dione) **15** 4
Bis(oxazolidine dione) **16** 4
Bis(oxazolidine dione) **17** 5
Assignment of Relative Stereochemistry for Bis(Triazoline) **10a** 5

Spectra of compounds 10a, 11c, 12a, 13a, 14a, 15–17 **6**

General Experimental Details. Flame-dried (under vacuum) glassware was used for all reactions. All reagents and solvents were commercial grade and purified prior to use when necessary. Acetonitrile (MeCN) and tetrahydrofuran (THF) were dried by passage through a column of activated alumina as described by Grubbs.[1] Thin layer chromatography (TLC) was performed using glass-backed silica gel (250 µm) plates and flash chromatography utilized 230–400 mesh silica gel from Sorbent Technologies. UV light, and/or the use of ceric ammonium molybdate and potassium iodoplatinate solutions were used to visualize products. IR spectra were recorded on a Nicolet Avatar 360 or a Thermo Electron (Nicolet) IR100/IR200 spectrophotometer and are reported in wavenumbers (cm^{-1}). Liquids and oils were analyzed as neat films on a NaCl plate (transmission), whereas solids were applied to a diamond plate (ATR) if a thin film could not be prepared.[2] Nuclear magnetic resonance spectra (NMR) were acquired on a Bruker instrument: AV-400 (400 MHz), DRX-500 (500 MHz), or AVII-600 (600 MHz). Chemical shifts are measured relative to residual nondeuterated solvent peaks as an internal standard set to δ 7.26 and δ 77.0 ($CDCl_3$) for ^1H and ^{13}C, respectively. Multiplicities are reported as singlet (s), doublet (d), triplet (t), quartet (q) or combinations thereof while higher coupling patterns are not abbreviated. High resolution mass spectra were recorded using a a Kratos MS-80 spectrometer by use of chemical ionization (CI) or electrospray ionization (ESI) at Indiana University. A post-acquisition gain correction was applied using sodium formate or sodium iodide as the lock mass. Regioselection of the (3+2) cycloaddition has been shown to favor the β-aminoxoazolidine dione in our prior work.[3]

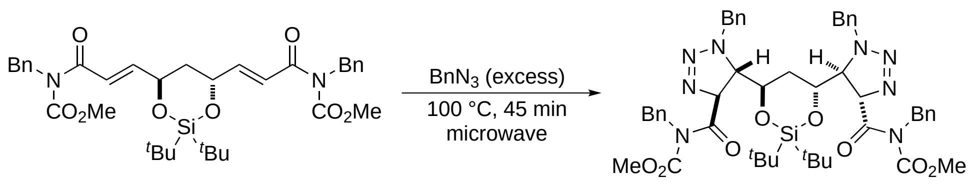

Triazoline 10. A solution of the bis(imide) (80.1 mg, 123 µmol) in benzyl azide (300 µL, 2.76 mmol) was subjected to 100 °C for 45 min in a Biotage microwave.[4] The mixture was directly purified using flash chromatography (SiO_2, 30 % ethyl acetate in hexanes) to afford the mixture of triazolines (1:2:1) as a yellow oil (81.6 mg, 72 %).
Data for the 2,3-*anti*/2′,3′-*anti* bis(triazoline) 10a. $[\alpha]_D^{20}$ −44.0 (*c* 0.50, $CHCl_3$); R_f = 0.27 (25% EtOAc/hexanes); IR (film) 3384, 3088, 3064, 3032, 2957, 2933, 2894, 2859, 1746, 1687 cm^{-1}; ^1H NMR (600 MHz, $CDCl_3$) δ 7.25–7.11 (m, 10H); 5.92 (d, *J* = 10.2 Hz,1H); 5.10 (d, *J* = 15.0 Hz, 1H); 4.88 (d, *J* = 15.0 Hz, 1H); 4.82 (d, *J* = 15.0 Hz, 1H); 4.72 (d, *J* = 15.0 Hz, 1H); 4.15 (dd, *J* = 13.2, 6.0 Hz, 1H); 4.00 (dd, *J* = 10.2, 7.8 Hz, 1H); 3.78 (s, 3H); 1.57 (t, *J* = 5.6 Hz, 1H); 0.92 (s, 9H); ^{13}C NMR (150 MHz, $CDCl_3$) ppm 169.5, 154.9, 136.8, 136.1, 128.4, 128.0, 127.5, 127.3, 127.2, 83.0, 72.5, 62.0, 54.1, 54.0, 48.4, 32.2, 27.0, 21.2; HRMS (CI): Exact mass calcd for $C_{49}H_{61}N_8O_8Si$ $[M+H]^+$ 917.4382, found 917.4404.

General Procedure for Triflic Acid-Promoted Bis(triazoline) Formation To a solution of the bisimide (1 equiv.) and benzyl azide (10 equiv.) in acetonitrile (0.15 M) at −20 °C was added triflic acid (5 equiv.) via syringe and the solution was stirred at −20 °C for 18 h. The reaction was quenched with triethylamine (5 equiv) via syringe and the reaction mixture was warmed to rt. The reaction mixture was poured into water and extracted with ethyl acetate. The combined organic layers were washed with brine, dried and concentrated. The resulting brown oil was purified using flash chromatography to afford the product.

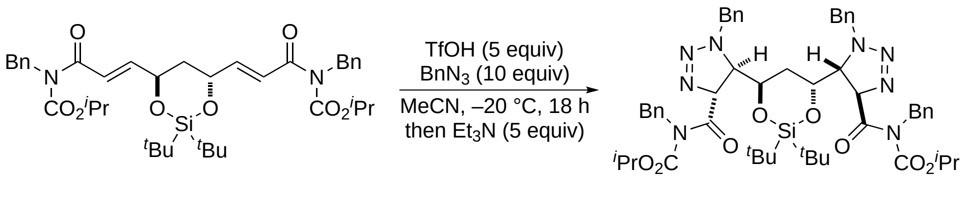

Triazoline 11. The imide (48.4 mg, 68.5 µmol) was treated with benzyl azide (91.1 mg, 685 µmol) according to the general procedure and purified using flash column chromatography (SiO_2, 30 % ethyl acetate in hexanes) to afford the triazoline mixture (1:9:9) as a yellow oil (36.0 mg, 54 %).

Data for the 2,3-*syn*/2′,3′-*syn* bis(triazoline) 11c. $[\alpha]_D^{20}$ +92.8 (*c* 0.75, $CHCl_3$); R_f = 0.20 (50% Et_2O/hexanes); IR (film) 3064, 3032, 2932, 2858, 1740, 1692 cm^{-1}; ^{1}H NMR (600 MHz, $CDCl_3$) δ 7.31–7.12 (m, 10H); 6.40 (d, *J* = 9.6 Hz, 1H); 5.03 (sept, *J* = 6.4 Hz, 1H); 5.00 (d, *J* = 15.4 Hz, 1H); 4.90 (d, *J* = 15.2 Hz, 1H); 4.86 (d, *J* = 15.2 Hz, 1H); 4.58 (d, *J* = 15.6 Hz, 1H); 4.17 (ddd, *J* = 10.4, 5.1, 5.1 Hz, 1H); 3.85 (dd, *J* = 9.1, 4.7 Hz, 1H); 1.54 (t, *J* = 5.6 Hz, 1H); 1.26 (d, *J* = 6.3 Hz, 3H); 1.17 (d, *J* = 6.3 Hz, 3H); 0.92 (s, 9H); ^{13}C NMR (150 MHz, $CDCl_3$) ppm 169.9, 153.7, 137.2, 135.6, 128.7, 128.4, 128.0, 127.8, 127.5, 127.3, 82.0, 71.9, 67.9, 62.5, 53.0, 48.2, 33.2, 27.0, 21.7, 21.4, 21.3; HRMS (ESI): Exact mass calcd for $C_{53}H_{69}N_8O_8Si$ [M+H]$^+$ 973.5008, found 973.4994.

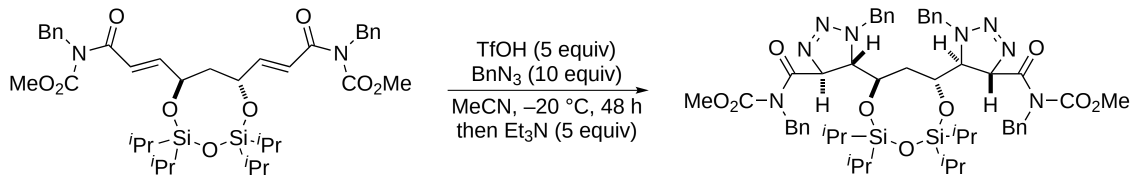

2,3-*anti*/2′,3′-*anti* Bis(triazoline) 13a. The imide (410 mg, 540 µmol) was treated with benzyl azide (665 mg, 5.00 mmol) according to the general procedure and purified using flash column chromatography (SiO_2, 25 % ethyl acetate in hexanes) to afford the triazoline mixture (18:1:1) as a yellow oil (150 mg, 27 %). $[\alpha]_D^{20}$ +56.9 (*c* 0.23, $CHCl_3$); R_f = 0.37 (25% EtOAc/hexanes); IR (film) 3031, 2945, 2866, 1740, 1691, 1496 cm^{-1}; ^{1}H NMR (600 MHz, $CDCl_3$) δ 7.26–7.18 (m, 8H); 7.10–7.08 (m, 2H); 6.08 (d, *J* = 10.2 Hz, 1H); 5.28 (d, *J* = 15.4 Hz, 1H); 4.95 (d, *J* = 15.2 Hz, 1H); 4.86 (d, *J* = 15.2 Hz, 1H); 4.49 (d, *J* = 15.4 Hz, 1H); 4.24 (ddd, *J* = 7.8, 5.9, 1.5 Hz, 1H); 3.82 (dd, *J* = 10.2, 1.4 Hz, 1H); 3.79 (s, 3H); 1.20 (dd, *J* = 8.1, 5.7 Hz, 1H); 1.07–1.08 (m, 6H); 1.01–0.96 (m, 4H); 0.90–0.85 (m, 4H); ^{13}C NMR (150 MHz, $CDCl_3$) ppm 169.9, 154.9, 137.0, 135.3, 128.6, 128.5, 128.0, 127.8, 127.3, 127.3, 81.3, 66.1, 62.3, 54.0, 52.7, 48.2, 36.2, 17.5 (2C), 17.4, 16.9, 13.9, 12.6; HRMS (ESI): Exact mass calcd for $C_{53}H_{70}N_8NaO_9Si_2$ [M+Na]$^+$ 1041.4702, found 1041.4757.

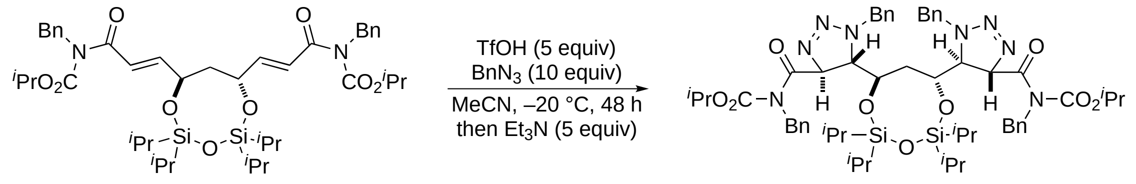

2,3-*anti*/2′,3′-*anti* Bis(triazoline) 14a. The imide (35 mg, 43 µmol) was treated with benzyl azide (57 mg, 430 µmol) according to the general procedure and purified using flash column chromatography (SiO_2, 25 % ethyl acetate in hexanes) to afford the triazoline mixture (18:1:1) as a yellow oil (34.9 mg, 75 %). $[\alpha]_D^{20}$ +132.9 (*c* 0.85, $CHCl_3$); R_f

= 0.50 (25% EtOAc/hexanes); IR (film) 3064, 3032, 2943, 2866, 1734, 1685, 1496 cm^{-1}; ^{1}H NMR (400 MHz, $CDCl_3$) δ 7.28–7.16 (m, 8H); 7.13–7.07 (m, 2H); 6.03 (d, *J* = 9.9 Hz, 1H); 5.32 (d, *J* = 15.6 Hz, 1H); 5.03 (septet, *J* = 6.3 Hz, 1H); 4.98 (d, *J* = 15.1 Hz, 1H); 4.82 (d, *J* = 15.1 Hz, 1H); 4.51 (d, *J* = 15.6 Hz, 1H); 4.33–4.25 (m, 1H); 3.83 (dd, *J* = 10.5, 1.5 Hz, 1H); 1.29 (d, *J* = 6.3 Hz, 3H); 1.23 (dd, *J* = 8.1, 5.8 Hz, 1H); 1.17 (d, *J* = 6.3 Hz, 3H); 1.12–0.80 (m, 14H); ^{13}C NMR (150 MHz, $CDCl_3$) ppm 170.0, 153.8, 137.3, 135.4, 128.6, 128.3, 128.0, 127.7, 127.4, 127.1, 81.2, 77.2, 71.9, 66.4, 62.2, 52.7, 48.1, 35.6, 21.8, 21.3, 17.5, 17.4 (2C), 16.8, 13.8, 12.5; HRMS (ESI): Exact mass calcd for $C_{57}H_{78}N_8NaO_9Si_2$ $[M+Na]^+$ 1097.5328, found 1097.5304.

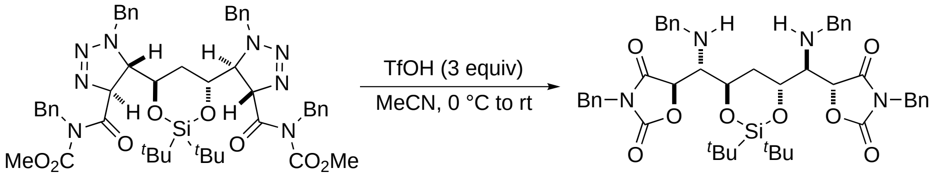

Bis(oxazolidine dione) 15. To a solution of triazoline (125 mg, 136 µmol) in acetonitrile (500 µL) at 0 °C was added triflic acid (26 µL, 300 µmol) via syringe. The solution was slowly warmed to rt and stirred for 15 h and then quenched with triethylamine (42 µL, 300 µmol). The reaction mixture was poured into water and extracted with ethyl acetate. The combined organic layers were washed with brine, dried and concentrated. The resulting yellow oil was purified using flash chromatography (SiO_2, 25 % ethyl acetate in hexanes) to afford the bis(oxazolidine dione) as a yellow oil (58 mg, 51 %) $[\alpha]_D^{20}$ −49.3 (*c* 1.5, $CHCl_3$); R_f = 0.30 (25% EtOAc/hexanes); IR (film) 3341, 3031, 2931, 2857, 1815, 1735, 1496 cm^{-1}; ^{1}H NMR (600 MHz, $CDCl_3$) δ 7.46–7.38 (m, 3H); 7.33–7.15 (m, 7H); 4.69 (d, *J* = 4.0 Hz, 1H); 4.68 (s, 2H); 4.49 (ddd, *J* = 11.0, 4.9 4.9 Hz, 1H); 3.91 (d, *J* = 12.6 Hz, 1H); 3.75 (d, *J* = 12.6 Hz, 1H); 3.07 (dd, *J* = 5.5, 4.1 Hz, 1H); 1.97 (dd, *J* = 5.8, 4.8 Hz, 1H); 1.92 (brs, 1H); 0.98 (s, 9H); ^{13}C NMR (150 MHz, $CDCl_3$) ppm 171.8, 155.2, 139.5, 134.6, 129.0, 128.9, 128.8, 128.73, 128.67, 128.6, 128.5, 128.4, 128.3, 127.2, 79.5, 69.7, 62.8, 53.6, 43.7, 33.9, 27.1, 21.2; HRMS (ESI): Exact mass calcd for $C_{47}H_{57}N_4O_8Si$ $[M+H]^+$ 833.3946, found 833.3946.

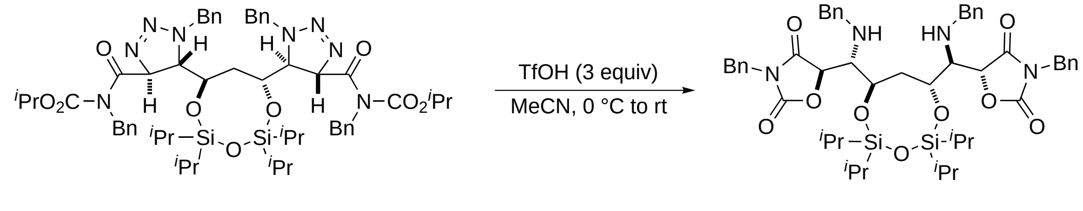

Bis(oxazolidine dione) 16. To a solution of triazoline (150 mg, 145 µmol) in acetonitrile (1.0 mL) at 0 °C was added triflic acid (40 µL, 450 µmol) via syringe. The solution was slowly warmed to rt and stirred for 10 h and then quenched with triethylamine (63 µL, 450 µmol). The reaction mixture was poured into water and extracted with ethyl acetate. The combined organic layers were washed with brine, dried and concentrated. The resulting yellow oil was purified using flash chromatography (SiO_2, 20 % ethyl acetate in hexanes) to afford the bis(oxazolidine dione) as a yellow oil (96 mg, 71 %). $[\alpha]_D^{20}$ +25.1 (*c* 0.71, $CHCl_3$); R_f = 0.38 (25% EtOAc/hexanes); IR (film) 3064, 3031, 2945, 2867, 1815, 1738, 1496 cm^{-1}; ^{1}H NMR (600 MHz, $CDCl_3$) δ 7.38–7.36 (m, 2H); 7.28–7.21 (m, 6H); 7.14–7.12 (m, 2H); 4.94 (d, *J* = 4.3 Hz, 1H); 4.68 (d, *J* = 14.5 Hz, 1H); 4.65 (d, *J* = 14.6 Hz, 1H); 4.31 (ddd, *J* = 7.5, 5.2, 5.2 Hz, 1H); 3.81 (d, *J* = 13.0 Hz, 1H); 3.73 (d, *J* = 13.0 Hz, 1H); 3.13 (d, *J* = 4.5, 4.6 Hz, 1H); 1.86 (dd, *J* = 7.6, 5.7 Hz, 1H); 1.09–0.98 (m, 14H); N−H was not observed; ^{13}C NMR (150 MHz, $CDCl_3$) ppm 171.4, 155.1,

139.4, 134.6, 129.0, 128.9, 128.8, 128.6, 128.6, 128.4, 128.3, 128.2, 127.2, 79.9, 68.0, 63.6, 53.2, 43.6, 36.8, 17.6, 17.5, 17.4, 17.2, 13.6, 12.8; HRMS (ESI): Exact mass calcd for $C_{51}H_{67}N_4O_9Si_2$ $[M+H]^+$ 935.4446, found 935.4454.

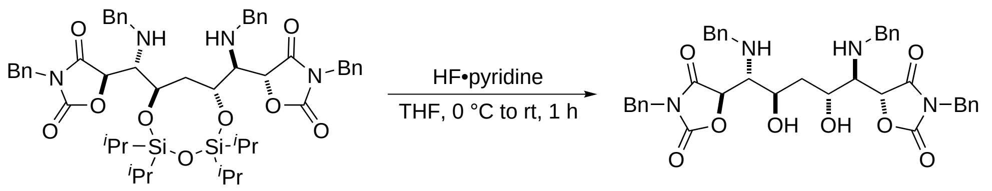

Bis(oxazolidine dione) 17. To a solution of the bis(oxazolidine dione) (100 mg, 107 µmol) in tetrahydrofuran (500 µL) was added HF–pyridine complex (65 µL) and the reaction was stirred at rt for 1 h. The reaction mixture was poured into water and extracted with dichloromethane. The combined organic layers were dried, concentrated and purified using flash chromatography (SiO_2, 50–70 % ethyl acetate in hexanes) to afford the diol as a colorless oil (28 mg, 38 %). $[\alpha]_D^{20}$ +30.0 (*c* 0.54, $CHCl_3$); R_f = 0.45 (50% EtOAc/hexanes); IR (film) 3482, 3031, 2924, 1811, 1732 cm^{-1}; ^1H NMR (400 MHz, $CDCl_3$) δ 7.38–7.34 (m, 2H); 7.28–7.22 (m, 6H); 7.18–7.13 (m, 2H); 4.95 (d, *J* = 2.0 Hz, 1H); 4.61 (d, *J* = 15.2 Hz, 1H); 4.57 (d, *J* = 15.2 Hz, 1H); 3.81 (d, *J* = 12.5 Hz, 1H); 3.76 (ddd, *J* = 11.0, 5.7, 5.7 Hz, 1H); 3.67 (d, *J* = 12.8 Hz, 1H); 3.05 (dd, *J* = 9.1, 2.0 Hz, 1H); 1.80 (dd, *J* = 5.6, 5.3 Hz, 1H); 1.70 (brs 2H); ^{13}C NMR (100 MHz, $CDCl_3$) ppm 172.5, 155.9, 138.6, 134.5, 128.7, 128.7, 128.6, 128.5, 128.2, 127.5, 79.3, 68.9, 62.4, 52.4, 43.7, 41.0; HRMS (ESI): Exact mass calcd for $C_{39}H_{41}N_4O_8$ $[M+H]^+$ 693.2924, found 693.2953.

Assignment of Relative Stereochemistry for Bis(Triazoline) 10a

Structural assignment of the triazolines was accomplished using 2D NMR (NOESY). Beginning at C1 next to the amide functionality, crosspeaks for H1/H2, H1/H3, H1/H4, H2/H3, H2/H6, and H3/H6 defined the orientation of all hydrogens and single bond rotations (Figure 1). No H1/H6 correlation was observed, supporting the rotamer depicted wherein this hydrogen is oriented away from H1. In contrast, the *syn–anti* diastereomer is not consistent with the observed crosspeaks. First, to support crosspeaks for H2/H6 and H3/H6, the *syn–anti* diastereomer should have an H4/H6 correlation, which was not observed. Additionally, single bond rotation to avoid this H4/H6 correlation gave opposite orientation between H2 and both of H3 which was not consistent with observed crosspeak between H2 and H3.

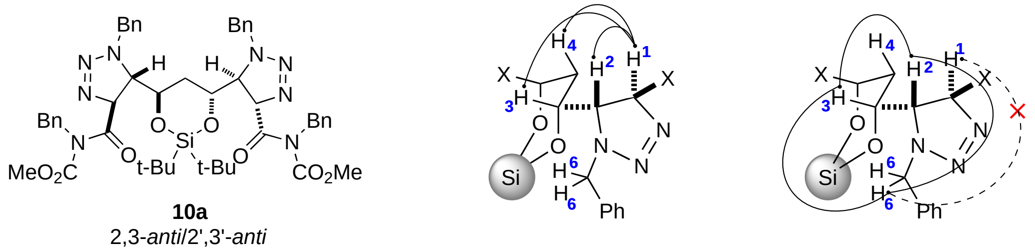

Figure 1. Diagnostic NOESY correlations for 2,3-*anti*/2′,3′-*anti* bis(triazoline) **10a**

References

[1] Pangborn, A. B.; Giardello, M. A.; Grubbs, R. H.; Rosen, R. K.; Timmers, F. J. *Organometallics* **1996**, *15*, 1518–1520.

[2] Note that triazoline N=N stretches are very weak when observed.

[3] (a) Mahoney, J. M.; Smith, C. R.; Johnston, J. N. *J. Am. Chem. Soc.* **2005**, *127*, 1354–1355; (b) Hong, K. B.; Donahue, M. G.; Johnston, J. N. *J. Am. Chem. Soc.* **2008**, *130*, 2323–2328; (c) Donahue, M. G.; Hong, K. B.; Johnston, J. N. *Bioorg. Med. Chem. Lett.* **2009**, *19*, 4971–4973.

[4] Caution should always be used when azides are heated or treated with strong acid, but we have never observed an explosion during our studies.

Spectra of compounds 10a, 11c, 12a, 13a, 14a, 15–17

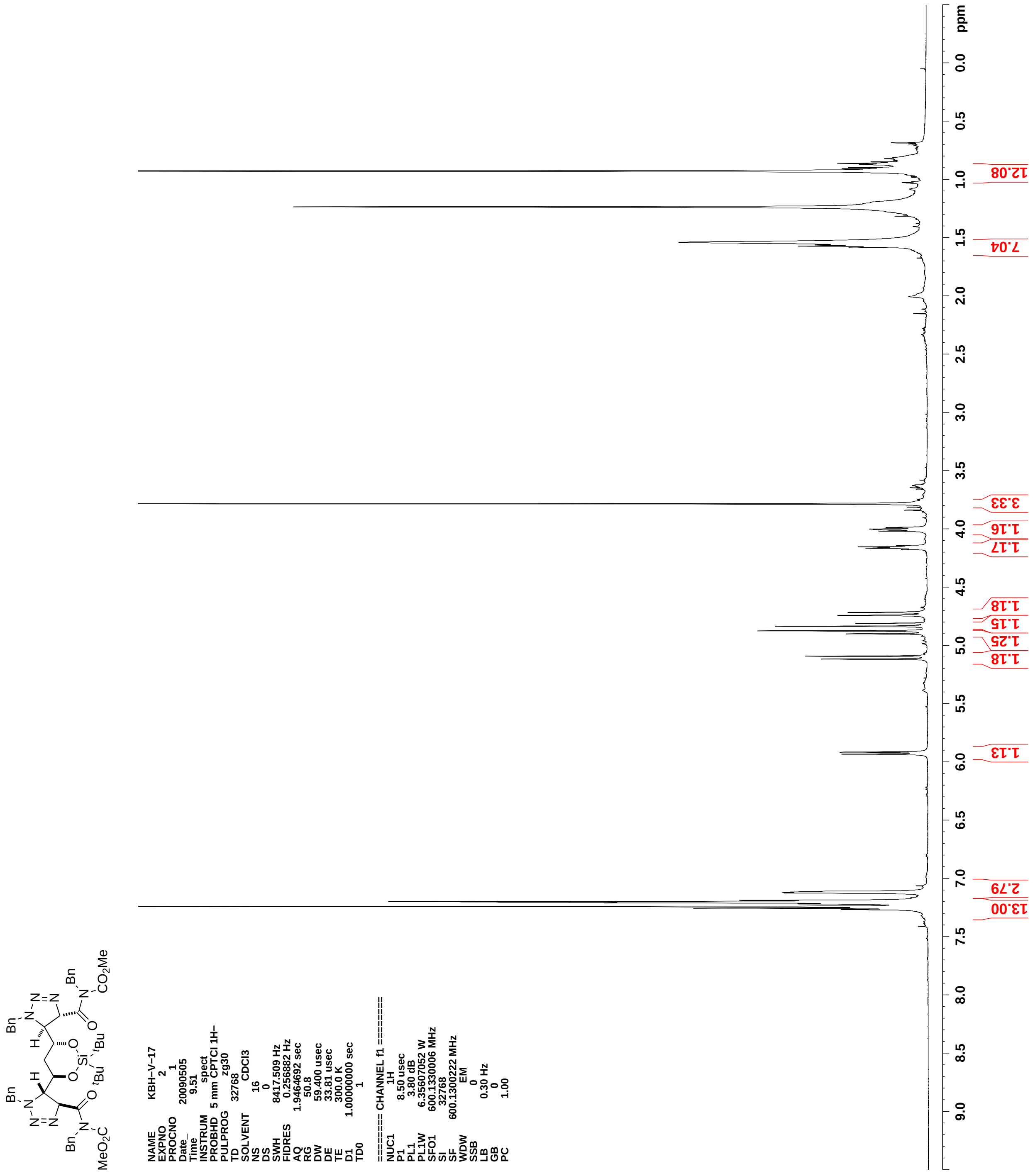

Figure 2. ^{1}H NMR ($CDCl_3$) of **10a**

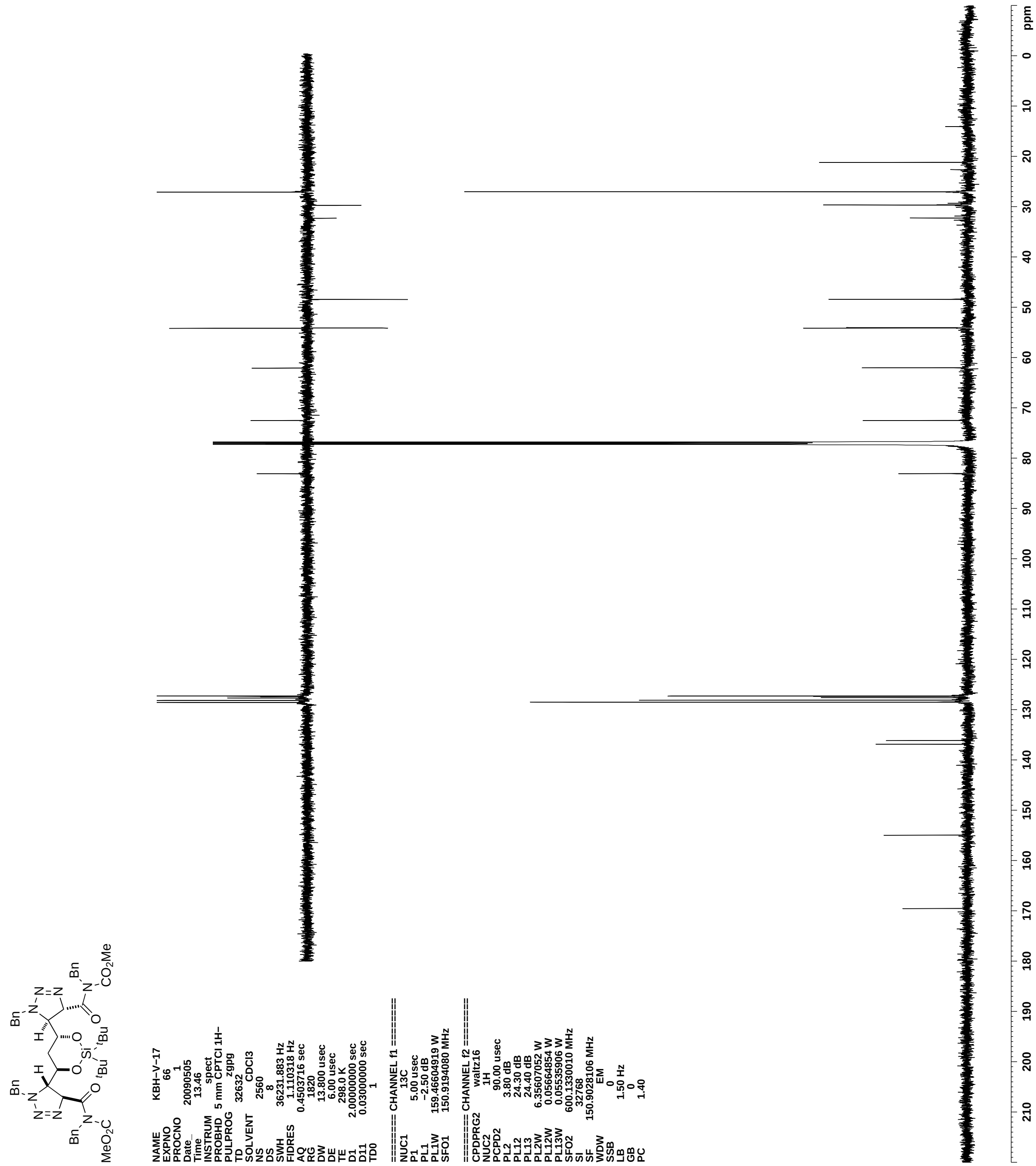

Figure 3. DEPT135 NMR (top) and ^{13}C NMR (bottom) ($CDCl_3$) of **10a**

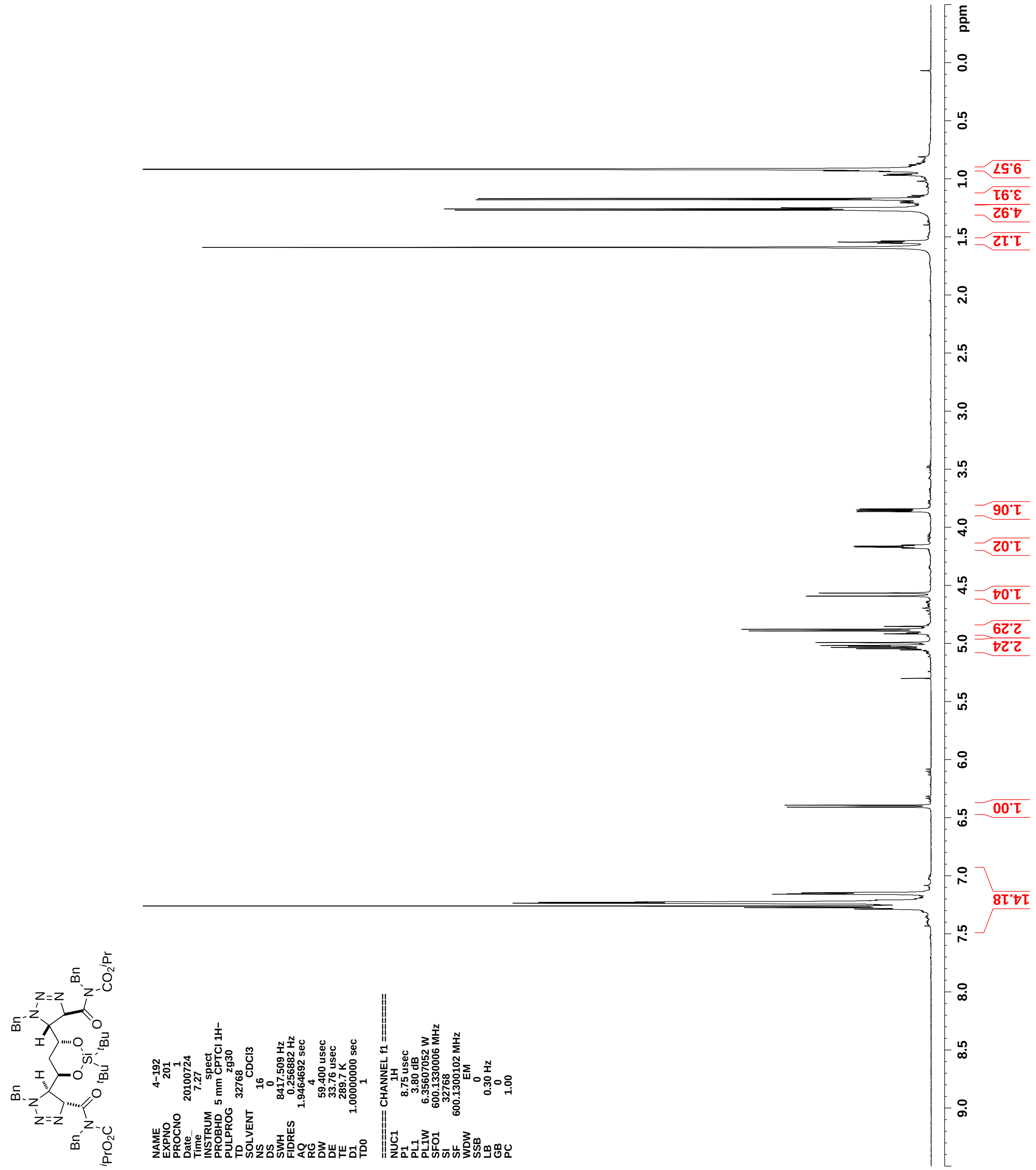

Figure 4. ^{1}H NMR ($CDCl_3$) of **11c**

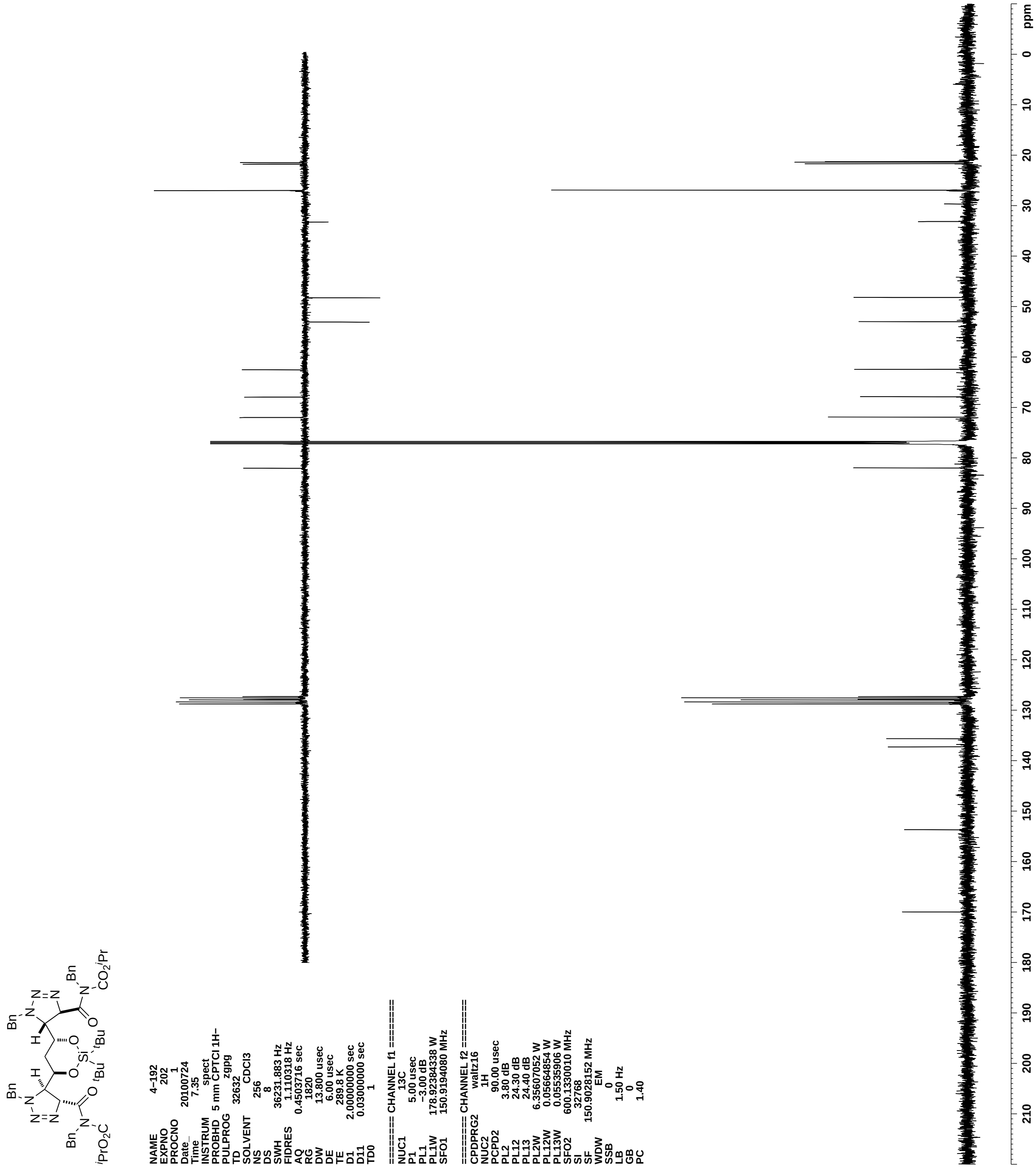

Figure 5. DEPT135 NMR (top) and ^{13}C NMR (bottom) ($CDCl_3$) of **11c**

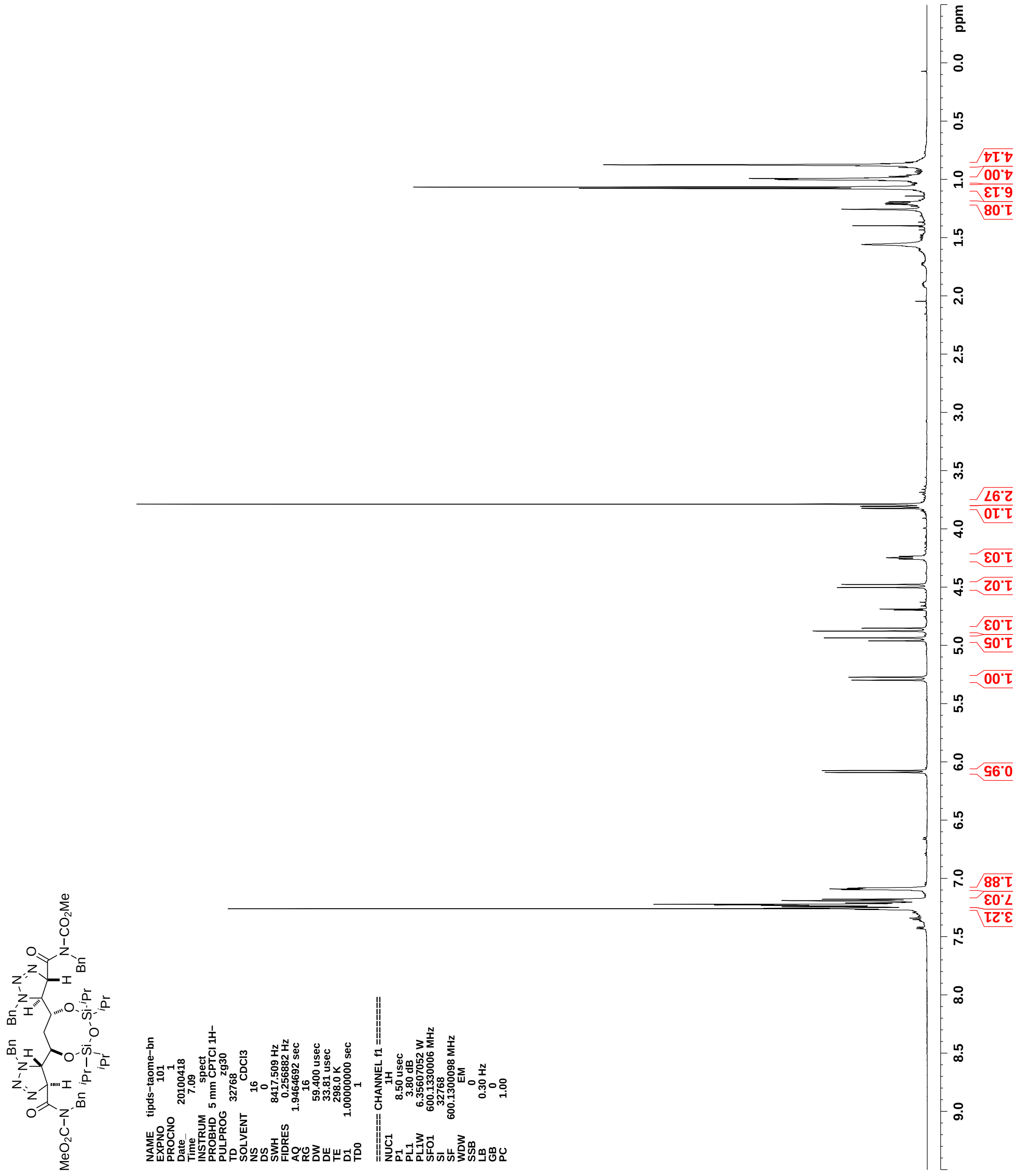

Figure 6. ^{1}H NMR ($CDCl_3$) of **13a**

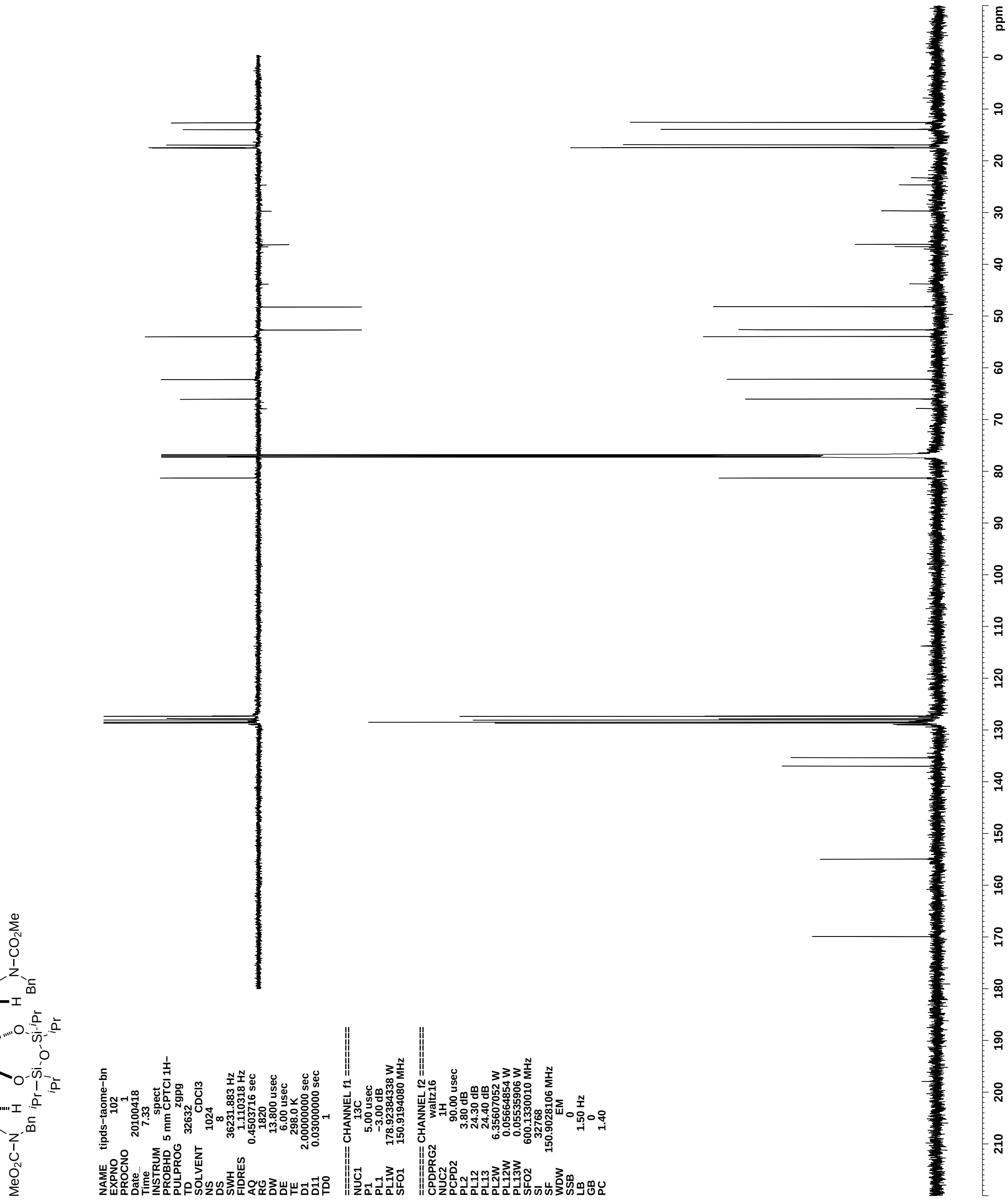

Figure 7. DEPT135 NMR (top) and ^{13}C NMR (bottom) ($CDCl_3$) of **13a**

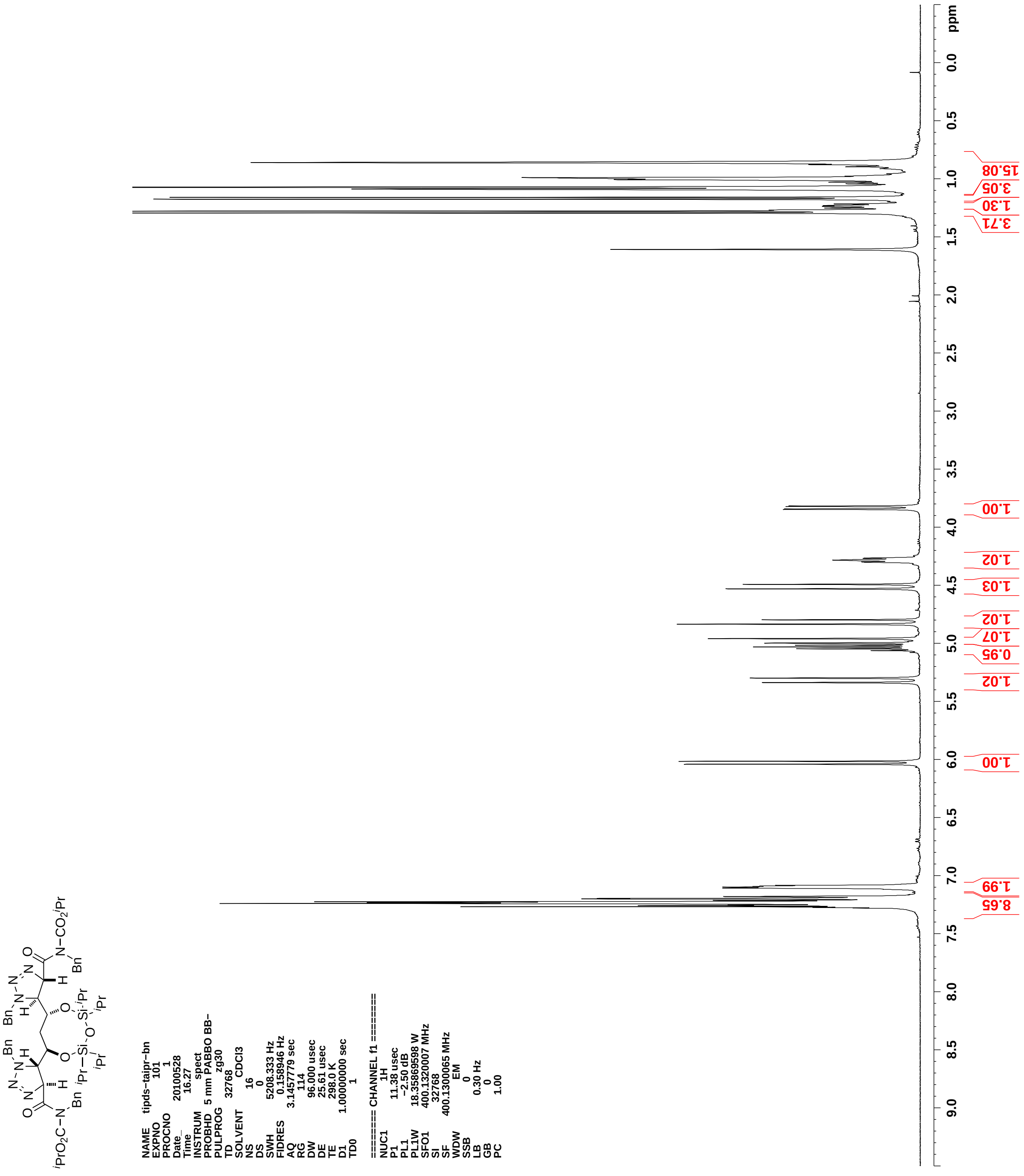

Figure 8. ^{1}H NMR ($CDCl_3$) of **14a**

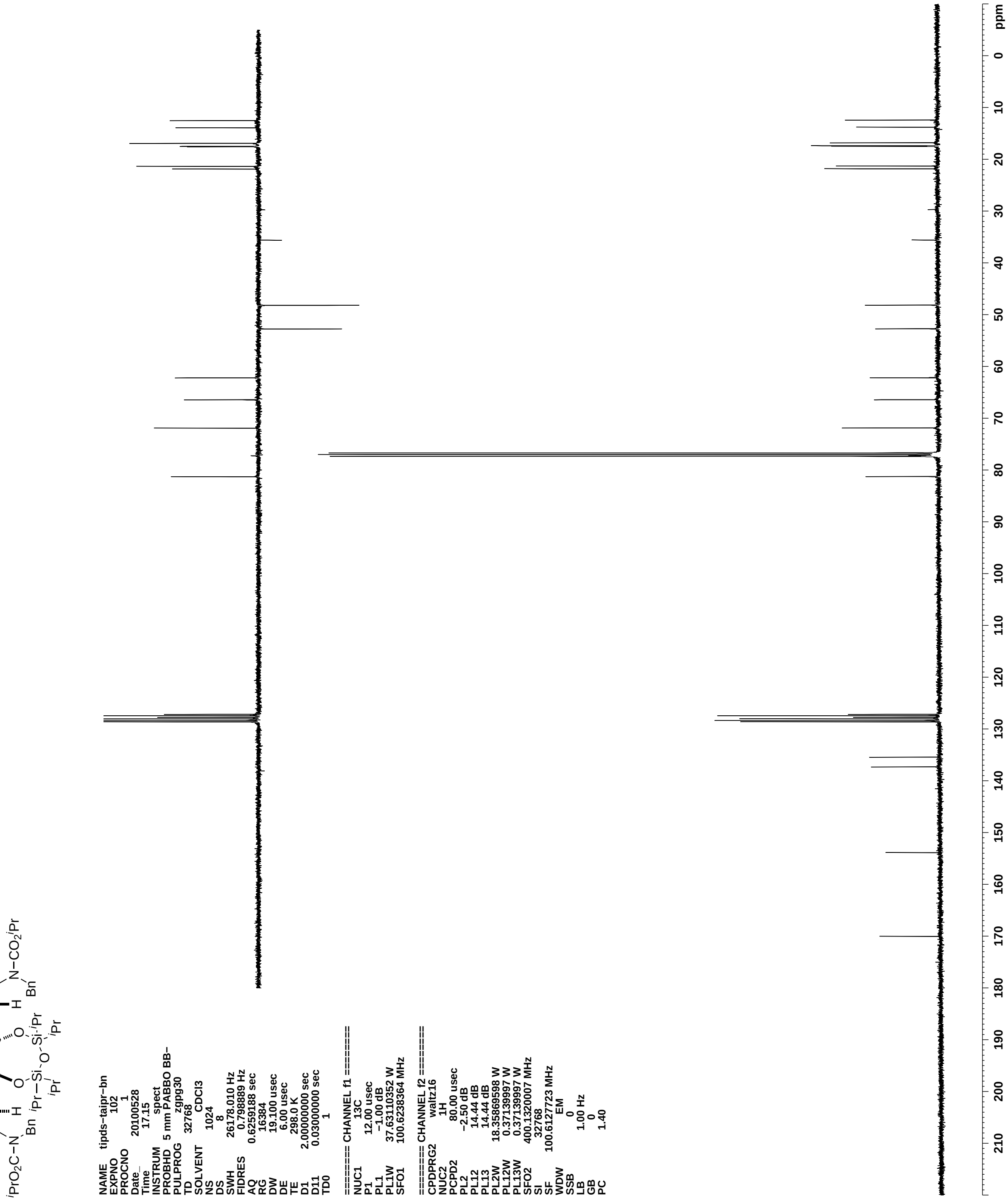

Figure 9. DEPT135 NMR (top) and ^{13}C NMR (bottom) ($CDCl_3$) of **14a**

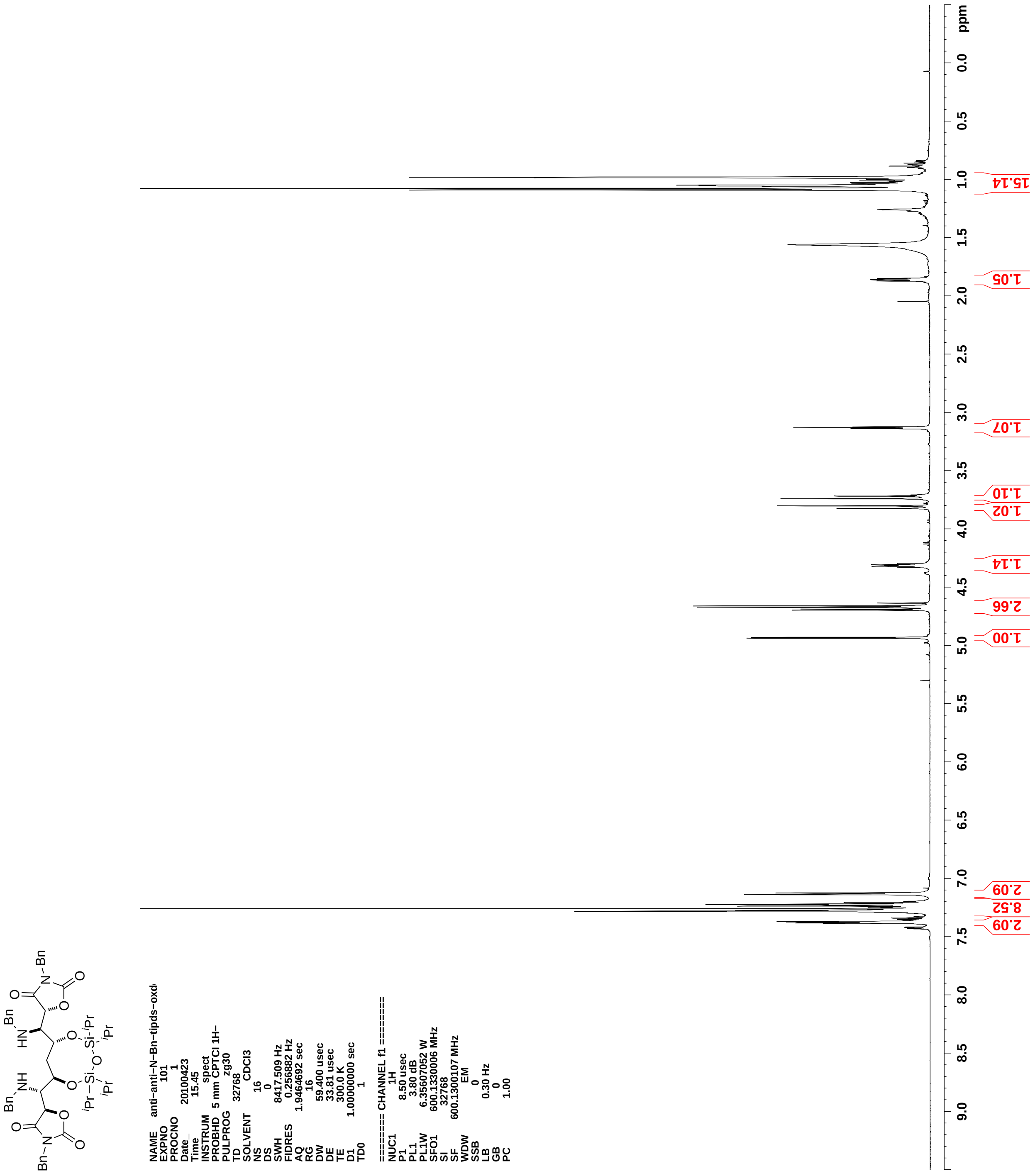

Figure 10. ^{1}H NMR ($CDCl_3$) of **15**

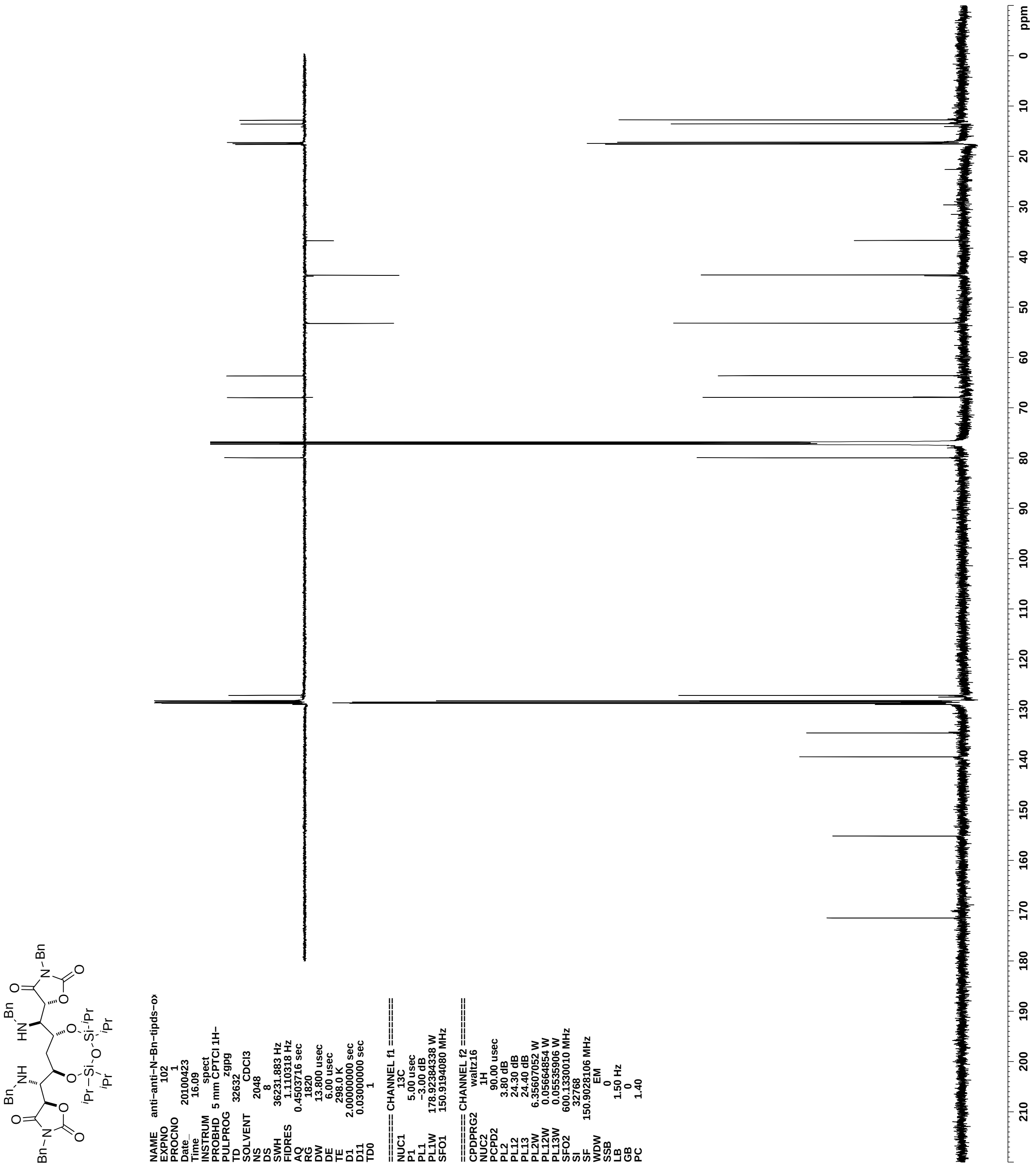

Figure 11. DEPT135 NMR (top) and ^{13}C NMR (bottom) ($CDCl_3$) of **15**

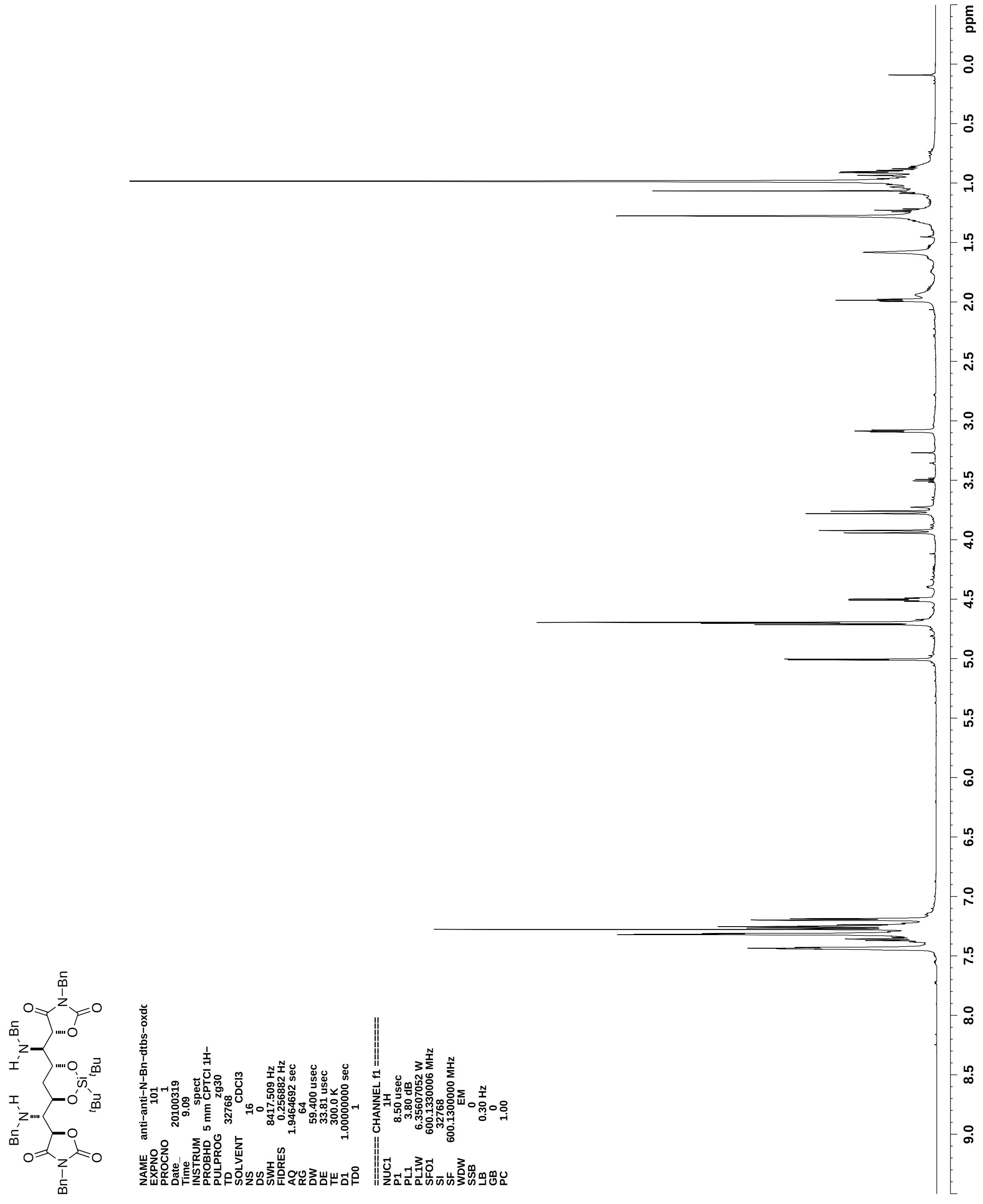

Figure 12. ^{1}H NMR ($CDCl_3$) of **16**

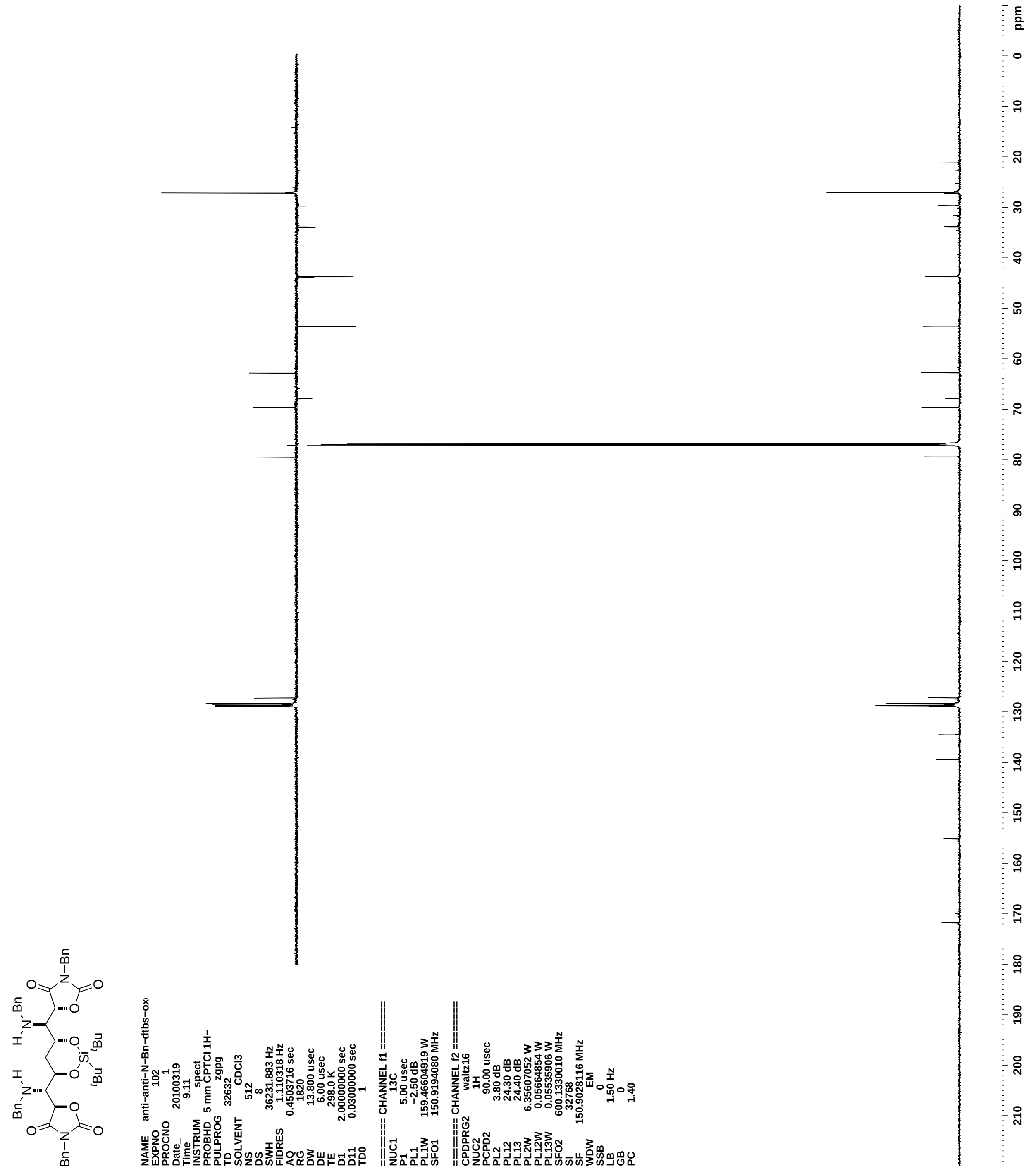

Figure 13. DEPT135 NMR (top) and ^{13}C NMR (bottom) ($CDCl_3$) of **16**

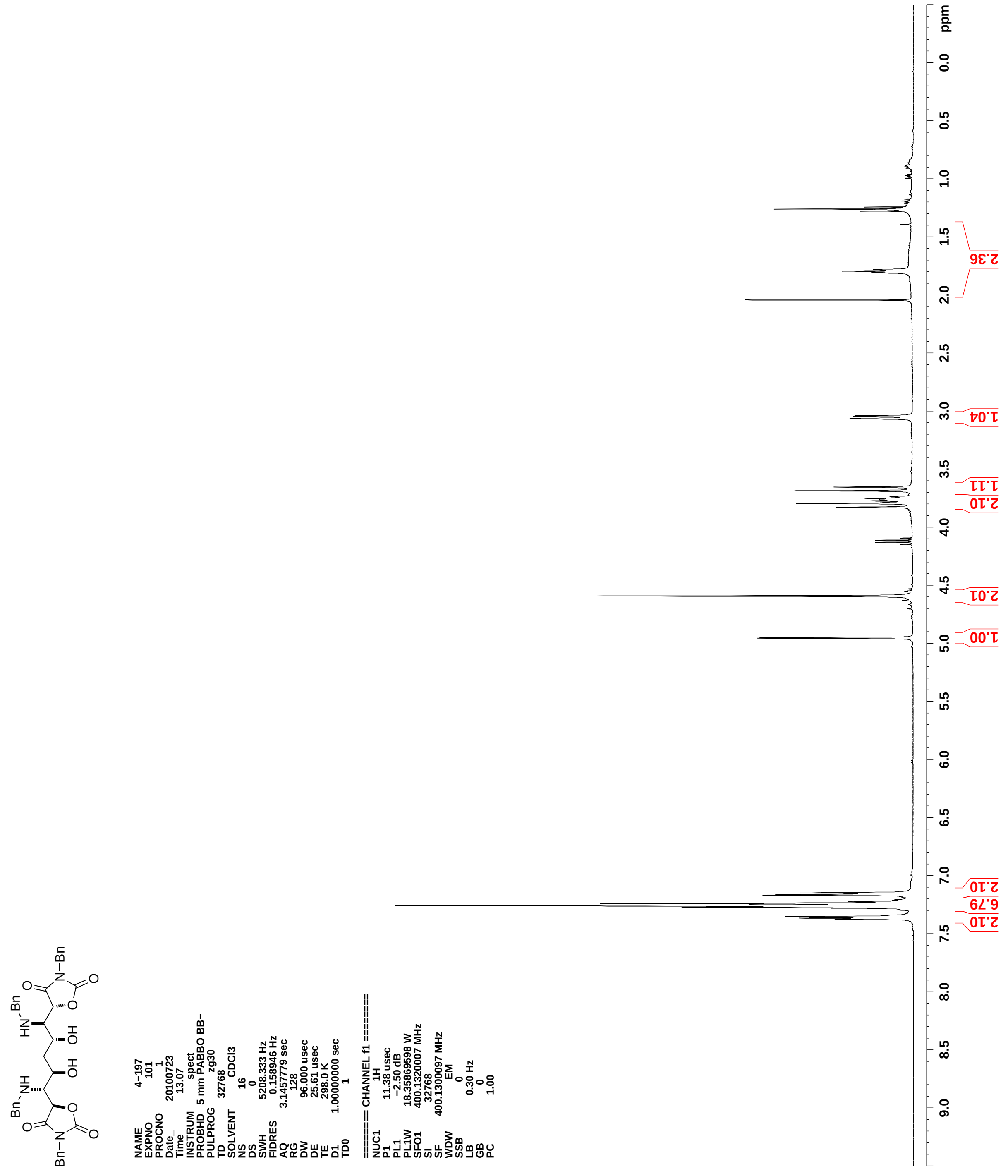

Figure 14. ^{1}H NMR ($CDCl_3$) of **17**

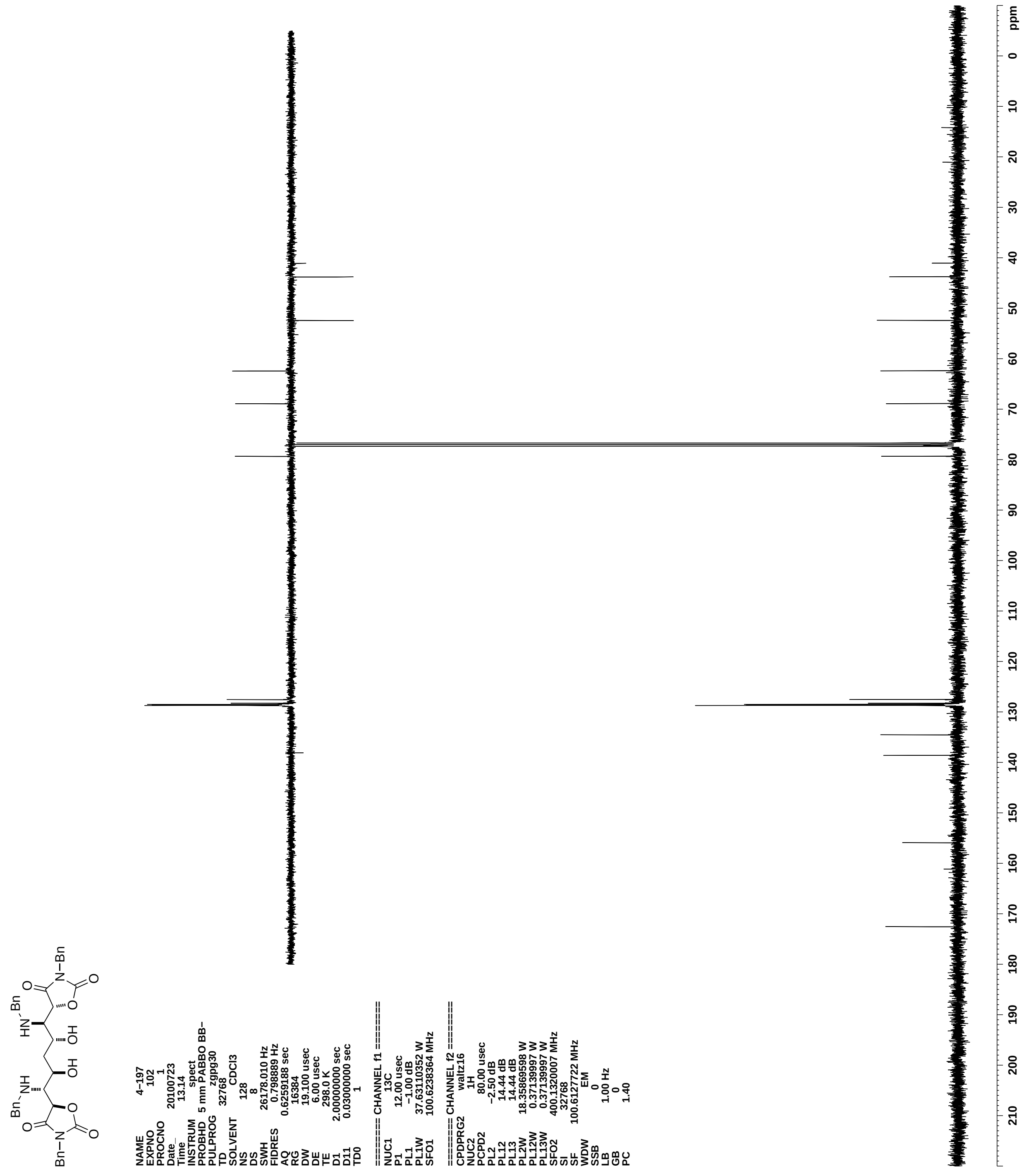

Figure 15. DEPT135 NMR (top) and ^{13}C NMR (bottom) ($CDCl_3$) of **17**
